# Enhanced mixing efficiency and reduced droplet size with novel droplet generators

**DOI:** 10.1038/s41598-024-55514-7

**Published:** 2024-02-27

**Authors:** Ali Kheirkhah Barzoki

**Affiliations:** Department of Mechanical Engineering, Sharif University of Technology, Tehran, Iran

**Keywords:** Microfluidics, Microchannel, Lab-on-a-chip, Mixing index, Mixing efficiency, Droplet, Droplet-generator, Two-phase, Finite element method (FEM), Mechanical engineering, Fluid dynamics

## Abstract

Nowadays, droplet microfluidics has become widely utilized for high-throughput assays. Efficient mixing is crucial for initiating biochemical reactions in many applications. Rapid mixing during droplet formation eliminates the need for incorporating micromixers, which can complicate the chip design. Furthermore, immediate mixing of substances upon contact can significantly improve the consistency of chemical reactions and resulting products. This study introduces three innovative designs for droplet generators that achieve efficient mixing and produce small droplets. The T-cross and cross-T geometries combine cross and T junction mixing mechanisms, resulting in improved mixing efficiency. Numerical simulations were conducted to compare these novel geometries with traditional T and cross junctions in terms of mixing index, droplet diameter, and eccentricity. The cross-T geometry exhibited the highest mixing index and produced the smallest droplets. For the flow rate ratio of 0.5, this geometry offered a 10% increase in the mixing index and a decrease in the droplet diameter by 10% compared to the T junction. While the T junction has the best mixing efficiency among traditional droplet generators, it produces larger droplets, which can increase the risk of contamination due to contact with the microchannel walls. Therefore, the cross-T geometry is highly desirable in most applications due to its production of considerably smaller droplets. The asymmetric cross junction offered a 8% increase in mixing index and around 2% decrease in droplet diameter compared to the conventional cross junction in flow rate ratio of 0.5. All novel geometries demonstrated comparable mixing efficiency to the T junction. The cross junction exhibited the lowest mixing efficiency and produced larger droplets compared to the cross-T geometry (around 1%). Thus, the novel geometries, particularly the cross-T geometry, are a favorable choice for applications where both high mixing efficiency and small droplet sizes are important.

## Introduction

The growing interest in chemistry and biology has amplified the demand for Lab-on-a-chip (LOC) design and processing technology. Microfluidics technology, a crucial aspect of biomedicine and biotechnology, has been widely utilized in various fields such as chemical synthesis, drug delivery, cell separation, and diagnostics^1–5^. This is due to its remarkable benefits in improving mass transfer, reducing the need for large sample and reagent quantities, and minimizing time and energy consumption^6^.

Droplet-based microfluidic systems have gained significant attention within the field of microfluidics due to their ability to enable a wide range of functions. These systems have proven to be highly versatile, allowing for tasks such as biochemistry, single cell analysis, chemical synthesis, polymerase chain reaction (PCR), loop-mediated isothermal amplification (LAMP), and the production of specialized microparticles and nanoparticles^7–12^. The main benefit of droplet-based microfluidics is its ability to accurately encapsulate extremely small amounts of reaction components, ranging from femto- to nanoliters. This allows for quick mixing and minimal heat transfer within each droplet, leading to precise control over reaction conditions. Additionally, using droplets as reaction chambers with minimal variation in volume ensures a uniform environment for the reaction^13^. In many applications involving microfluidic systems, efficient mixing is crucial to their performance. However, due to their small size, the flow in these systems is typically laminar, which limits mixing to diffusion. As a result, achieving adequate mixing can be challenging^14,15^.

One method suggested to improve mixing in microfluidic devices is the use of micromixers, which can be either passive or active. Passive micromixers employ specific geometries or microstructures within the microfluidic channels to enhance the interaction between fluids and promote more efficient mixing^16–20^. In contrast, active micromixers utilize control elements that necessitate extra energy, such as electroosmotic, magnetic, and acoustofluidic mixing^21–25^. While passive mixers face difficulties in achieving high mixing efficiency, active mixers offer the advantage of more efficient mixing of reaction components. However, this comes at the expense of increased energy consumption and a more intricate device design. Numerous studies have focused on enhancing the mixing index in droplet-based microfluidic systems by incorporating micromixers directly after the droplet generator^26–32^. Implementing micromixers induce advection in the droplets, thereby improving mixing. However, it is important to consider that incorporating micromixers adds complexity to the chip, increases its size, and restricts the frequency of droplet generation due to increased hydrodynamic resistance^33^. As a result, there is a growing need for alternative mixing methods that do not rely on micromixers.

An alternative approach in mixing in the microfluidic chips involves using droplets to mix reagents during the droplet formation process. When two immiscible fluids come together at a junction, different forces like viscosity, surface tension, and pressure gradient interact. As a result, the droplet separates from the dispersed phase and continues to flow downstream. By maintaining a controlled droplet size, this technique prevents the axial dispersion of mixing components, enabling rapid mixing through the internal circulation of the droplet^34,35^. Belousov et al. developed an asymmetric flow focusing droplet generator that enhances mixing during the droplet formation stage and demonstrated a six-fold increase in mixing speed compared to symmetric design. It's important to note that flow focusing has its own advantages. It operates by compressing the dispersed phase by the continuous phase from two side channels leading to the formation of droplets. This design ensures that droplets do not come into contact with the channel walls, minimizing any potential contamination in the dispersed phase components. However, the T junction offers the advantage of more efficient mixing although the droplet will be larger and more in contact with the walls of the channel. Therefore, it is reasonable to consider modifying the flow focusing design to further improve mixing capabilities.

**Figure 1:**
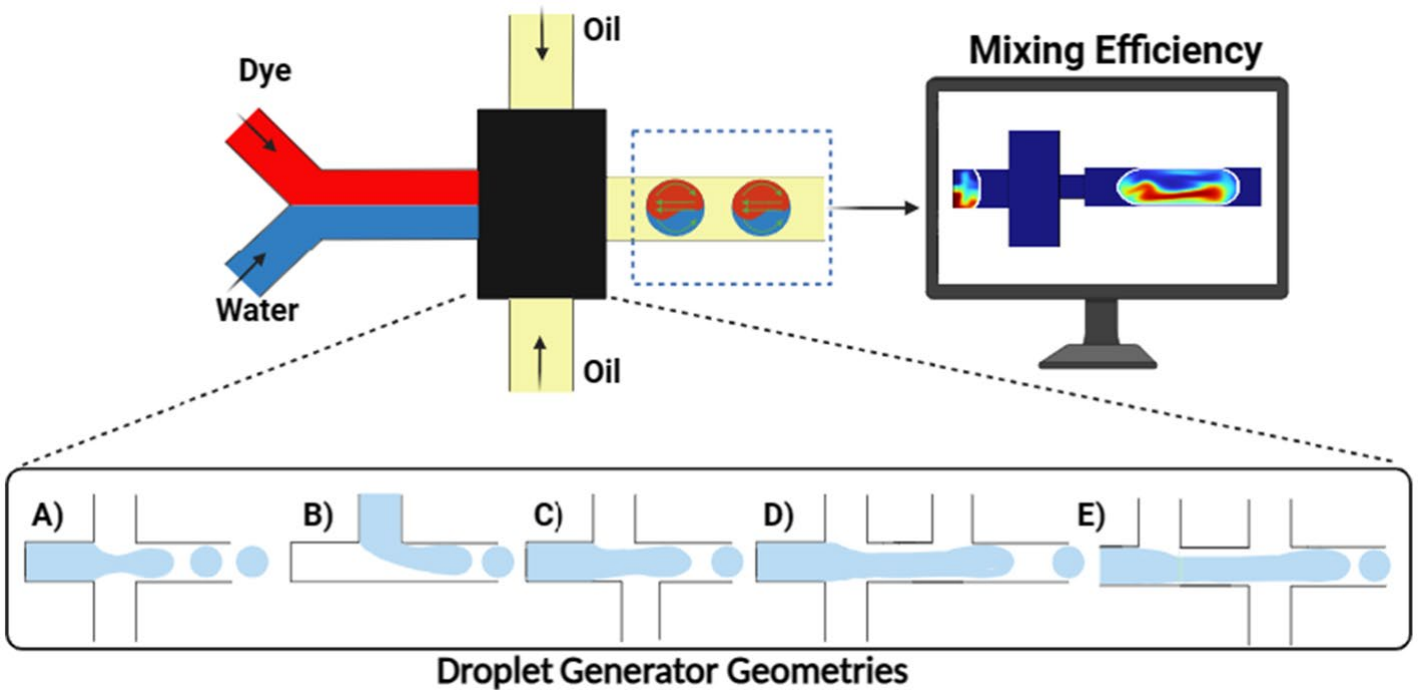
The schematic illustration of the study. There are five different geometries investigated: (**A**) cross junction, (**B**) T junction, (**C)** asymmetric cross junction, (**D**) cross-T junction, and (**E**) T-cross junction.

In this research, I propose three novel droplet generators based on the conventional flow-focusing and T junction geometries. In comparison to the cross junction and T junction, the modifications in these geometries provide higher mixing efficiencies while keeping the droplet size relatively small. A comprehensive study is performed to compare the mixing efficiency, droplet diameter, and eccentricity of the droplets in these geometries. The proposed novel geometries entail asymmetric cross junction, T-cross junction, and cross-T junction (see Fig. 1). The simulations demonstrated that these novel designs, by combining the mixing mechanisms of the T junction and cross junction, provide improved mixing efficiency with droplet diameter smaller than that in the T junction geometry. This study offers valuable insights into the selection of optimal designs to improve mixing efficiency in droplet-based systems.

## Methods

### Governing equations

Navier–Stokes equations were used to model the liquid mediums. Considering Newtonian and incompressible fluid in the laminar flow regime, these governing equations were utilized as in Eqs. (1) and (2), as the maximum fluid flow rate was kept at 10 μL/min $\left( {Re = \frac{\rho uw}{\mu } \approx 1} \right).$

1 $$ \nabla .{\vec{\text{u}}} = 0 $$

2 $$ \rho \frac{{\partial {\vec{\text{u}}}}}{\partial t} + \rho \left( {{\vec{\text{u}}} .\nabla } \right){\vec{\text{u}}} = - \nabla {\text{P}} + {\upmu }\nabla^{2} {\vec{\text{u}}} + \overrightarrow {{{\text{F}}_{s} }} $$

where ${\vec{\text{u}}}$ represents the velocity vector ($m/s$), P is the pressure ($Pa$), $\mu$ shows the dynamic viscosity ($Pa.s$), $\rho$ denotes the fluid density ($kg/m^{3}$), and $F_{s}$ is the surface tension force ($N/m^{3}$), which is solved using continuum surface force (CSF) model:

3 $$ \overrightarrow {{{\text{F}}_{s} }} = \sigma \frac{{\rho k\frac{{\vec{n}}}{{\left| {\vec{n}} \right|}}}}{{\frac{1}{2}\left( {\rho_{c} + \rho_{d} } \right)}} $$

4 $$ k = \nabla \cdot \frac{{\vec{n}}}{{\left| {\vec{n}} \right|}} $$

$\sigma$ represents the surface tension. The local surface curvature, denoted by k, is defined as the divergence of the normal vector $\vec{n}$ normalized by its magnitude. Subscripts c and d distinguish between the continuous phase and dispersed phase, respectively. $\rho$ and $\mu$ remain constant and denote the average density and average viscosity of the continuous and dispersed phases, respectively, which are calculated using Eqs. (5) and (6):

5 $$ \rho = \alpha_{c} \rho_{c} + \alpha_{d} \rho_{d} $$

6 $$ \mu = \alpha_{c} \mu_{c} + \alpha_{d} \mu_{d} $$

where $\alpha_{c}$ and $\alpha_{d}$ denote the volume fraction of the continuous and dispersed phases, respectively.

Due to variations in intermolecular forces between oil, water, and channel wall, a contact angle denoted as θ emerges at the interface of the oil–water and solid surfaces when they come into contact. This contact angle is taken into account through the utilization of the contact angle model^36^. Within the CSF model framework, the impact of the assumed contact angle is addressed by modifying the curvature of the fluid interface in the meshes near the channel walls. This adjustment is mathematically expressed by the following equation:

7 $$ \vec{n} = \vec{n}_{w} \cos \theta + \vec{t}_{w} \sin \theta $$

Here, $\vec{n}_{w}$ and $\vec{t}_{w}$ represent the normal and tangential vectors of the microchannel walls, respectively.

In order to track the fluid–fluid interface in our two-phase system, the Cahn–Hilliard equation was utilized^37^:

8 $$ \frac{\partial \phi }{{\partial t}} + {\vec{\text{u}}} \nabla \phi = \nabla . \left( {\frac{\gamma \lambda }{{\varepsilon^{2} }} \nabla \Psi } \right) $$

9 $$ \Psi = - \nabla .(\varepsilon^{2} \nabla \phi ) + \phi \left( {\phi^{2} - 1} \right) $$

In Eqs. (8) and (9), $$\phi$$ represents the phase variable ensuring a smooth transition across the phase interface; $$\varepsilon$$ shows the interfacial thickness, which was set to half of the maximum mesh size; $${\Psi }$$ is an auxiliary variable in order to reduce the differential equation order. In Eq. (8), $$\gamma$$ denotes the mobility parameter, which is proportional to the square of interface thickness $${\upgamma } = {\chi  }\varepsilon^{2}$$, where $${\upchi }$$ is the mobility tuning parameter, which was set to 1 in this study. $$\lambda$$ is the mixing energy density defined as $$\lambda = {\raise0.7ex\hbox{${3\sigma }$} \!\mathord{\left/ {\vphantom {{3\sigma } {\sqrt 8 \varepsilon }}}\right.\kern-0pt} \!\lower0.7ex\hbox{${\sqrt 8 \varepsilon }$}}$$, where $$\sigma$$ is the surface tension coefficient. In Eq. (2), by multiplying the chemical potential (G) by the gradient of the phase field ($$F_{s} = G\nabla \phi$$) the surface tension force could be determined. The definition of the chemical potential is as follows^38^:

10 $$ G = \lambda \left( { - \nabla_{t}^{2} \phi + \frac{{\phi \left( {\phi^{2} - 1} \right)}}{{\varepsilon^{2} }}} \right) $$

The main advantage of using the phase-field method to model two-phase flows is its capability to accurately calculate the displacement of the contact line while maintaining a no-slip boundary condition for fluid velocity. Moreover, it effectively eliminates pressure discontinuities at corners and prevents the formation of artificial vortices in intersecting channel regions. Additionally, as an interface capturing technique, it allows for precise resolution of droplet breakup.

In order to simulate the mixing process, the convective-diffusive mass transport equation was utilized:

11 $$ {\raise0.7ex\hbox{${\partial c}$} \!\mathord{\left/ {\vphantom {{\partial c} {\partial t}}}\right.\kern-0pt} \!\lower0.7ex\hbox{${\partial t}$}} + {\vec{\text{u}}} .\nabla c = {\text{D}}\nabla^{2} c $$

where D and c are the diffusion coefficient ($m^{2} /s$) and the dye concentration $\left( {mol/m^{3} } \right)$, respectively. To calculate the mixing index (MI), the dye concentration in each mesh cell was considered using the following formula^39^:

12 $$ MI \left( \% \right) = \left( {1 - \sqrt {\frac{{{\iint }\left( {c - \overline{c}} \right)^{2} dA}}{{A \cdot \overline{c }^{2} }}} } \right) \times 100 $$

where c, $\overline{c}$, and $c_{max}$ are the dye concentration, average and maximum dye concentration ($mol/m^{3}$) in the droplet. A denotes the droplet area ($m^{2}$).

It is worth mentioning that during this investigation, the characteristics of the dye solution were considered to be the same as those of DI water, which has a density of 1000 $kg/m^{3}$ and a dynamic viscosity of $1 mPa.s$. Furthermore, the simulations used properties for olive oil, with a density of 917 $kg/m^{3}$ and a dynamic viscosity of $84 mPa.s$. The surface tension was set at $23.6 mN/m$^40^. The diffusion coefficient value was set to $5.75 \times 10^{ - 10} m^{2} /s$. The boundary conditions used in the simulations can be found in Table 1, which includes $u_{n}$, $u_{t}$, $\mu$, V_interface_, and Q representing the normal and tangential velocities, dynamic viscosity, velocity of the interface, and flow rate, respectively. The velocity distribution at the inlets was considered parabolic.

**Table 1:** Boundary conditions.

	Boundary condition
Liquid–Liquid Interface	$u_{n} |_{in} = u_{n} |_{out} = V_{interface}$ $u_{t} |_{in} = u_{t} |_{out}$ $\mu_{in} \frac{{\partial u_{t} }}{\partial r}|_{in} = \mu_{out} \frac{{\partial u_{t} }}{\partial r}|_{out}$
Liquid–Solid interface	$u_{interface} = V_{interface} \left( {no slip} \right)$
inlets	$Q = constant$
Outlet	*P* = *0*

**Figure 2:**
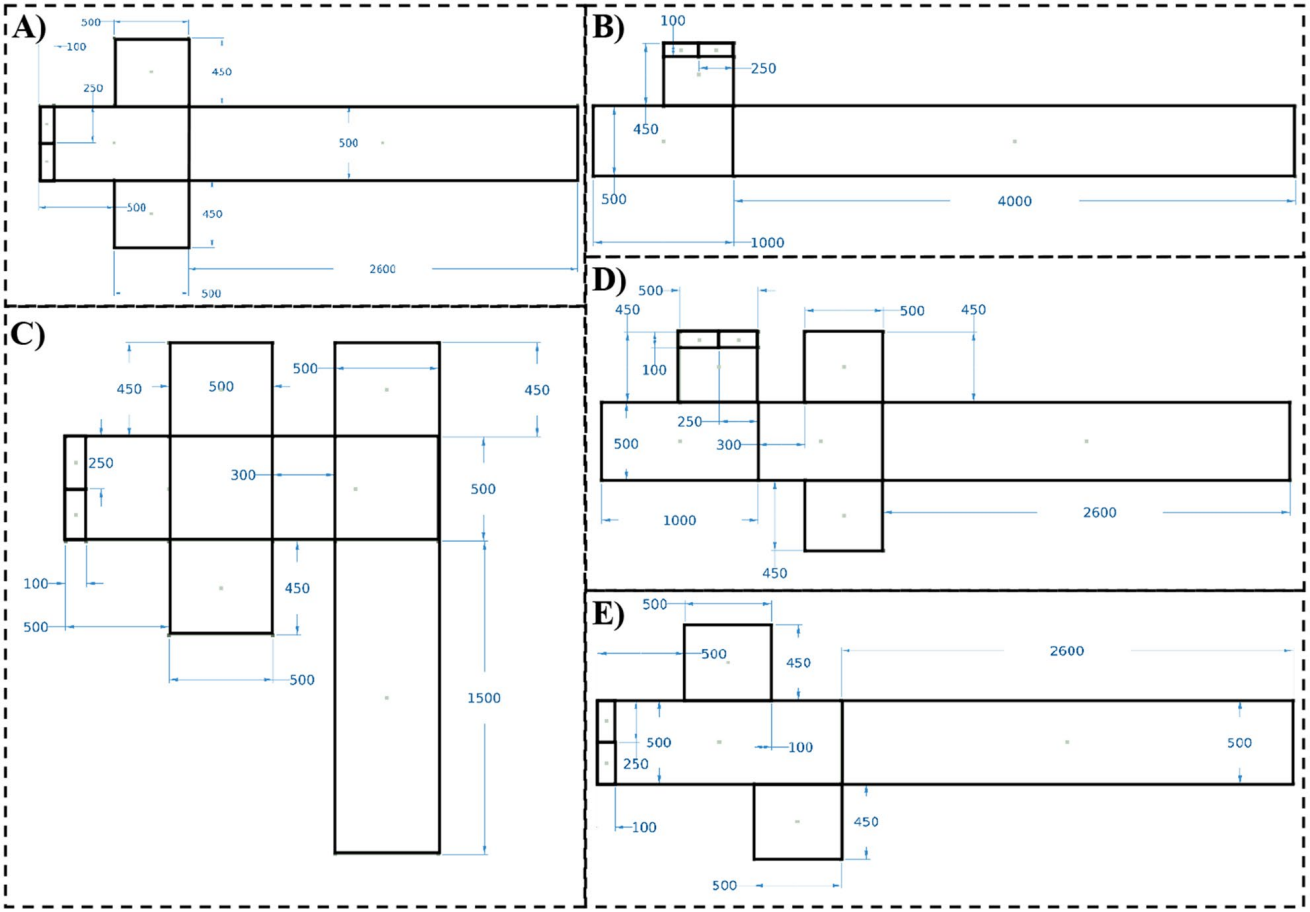
Droplet generator geometries. The dimensions of the (**A**) cross junction, (**B**) T junction, (**C**) cross-T, (**D**) T-cross, and (**E**) asymmetric cross junction. The dimensions are in $\mu m$.

To have a meaningful comparison between different geometries, the total flow rate of the continuous phase was considered $10 \mu l/min$. As a result, in geometries with two continuous phase inlets, like cross junction and asymmetric cross junction, each inlet has a flow rate of $5 \mu l/min$, while in geometries with three continuous phase inlets, like T-cross and cross-T geometries, each inlet has a flow rate of $3.33 \mu l/min$. In the T junction geometry, the continuous phase flow rate was considered $10 \mu l/min$. The geometries and dimensions are illustrated in Fig. 2.

### Computational method

Numerical simulations were conducted using the finite element method (FEM) to evaluate the mixing efficiency within droplets. Mixing efficiency assesses how evenly different analytes are distributed within a droplet. Three sets of equations needed to be simultaneously solved in order to determine the mixing efficiency. Parallel Direct Sparse Solver (PARDISO) with a residual tolerance of 1E-3 was implemented to solve the fluid flow and phase-field equations. The mixing equation was solved using the Multifrontal Massively Parallel Sparse Direct Solver (MUMPS) with left preconditioning and a residual tolerance set at 1E-3. The discretization of pressure and velocity involved the use of first-order and second-order elements, respectively. Linear discretization was applied to both the phase-field and mixing solutions. At each time step, the Newton method was utilized to linearize the set of non-linear equations. Since the simulations were unsteady, the time step ($\Delta \tau$) was established based on the Courant number (CFL < 0.3), given by $CFL = \left( {u\Delta \tau } \right)/\Delta h$, where u represents the flow velocity, and Δh denotes the mesh size. Triangular (tri) elements were used to discretize the entire domain, including the boundary layers. For the simulations in this study, 2D geometries were employed. While 3D geometries may provide the most precise outcomes, 2D geometries have proven to be useful in providing valuable information and showing acceptable consistency with experimental data, all without the need for extensive computational power^33,41–43^.

### Mesh independence test

The size of mesh elements is a crucial factor in numerical analysis, impacting the accuracy of results and computation time. To achieve optimal mesh size, a mesh independence test was conducted, varying element sizes from 7 to 52.5 $\mu m$. The test results in Table 2 demonstrate that the mixing index experiences an enhancement when the element size falls below 17.5 $\mu m$. Despite similar results for 17.5, 14, and 7 $\mu m$, the choice of 14 $\mu m$ was preferred due to both shorter computational time and more accurate results. Therefore, a mesh element size of 14 $\mu m$ was adopted for the subsequent simulations. To be noted that this study was performed for the cross-T geometry with a flow rate ratio of 0.5.

**Table 2:** Mesh independence study.

Mesh element size in $\user2{\mu m}$	Number of mesh elements	Mixing index (%)
52.5	1315	63.64
35	5281	89.28
24.5	10,581	81.03
17.5	20,317	76.51
14	31,170	76.28
7	124,374	76.32

### Model validation

**Figure 3:**
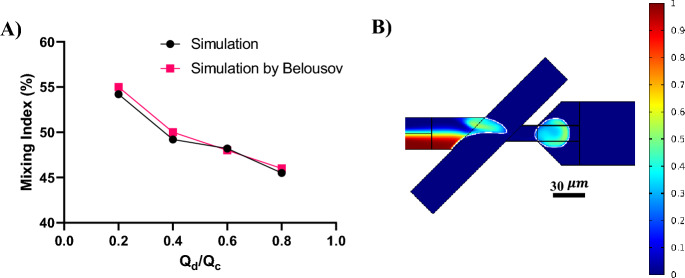
Validation of the simulations in different flow rate ratios. (**A**) Comparative analysis of the mixing index and (**B**) Simulation of the droplet formation and mixing for the flow rate ratio of 0.2. The continuous phase flow rate (Q_c_) is $1 \mu l/min$.

To verify the accuracy of the simulations, the model was validated by the results of the research by Belousov et al^33^. The mixing index in different flow rate ratios of the dispersed phase to the continuous phase (Q_d_/Q_c_) were compared. The result is illustrated in Fig. 3. The asymmetric geometry with a depth of 40 $\mu m$ was considered for the validation. The simulations show a proper agreement with the results by Belousov et al. One of the simulations of the validation is depicted in the Fig. 3b.

## Results and discussion

### Fluid dynamics of the droplet formation

**Figure 4:**
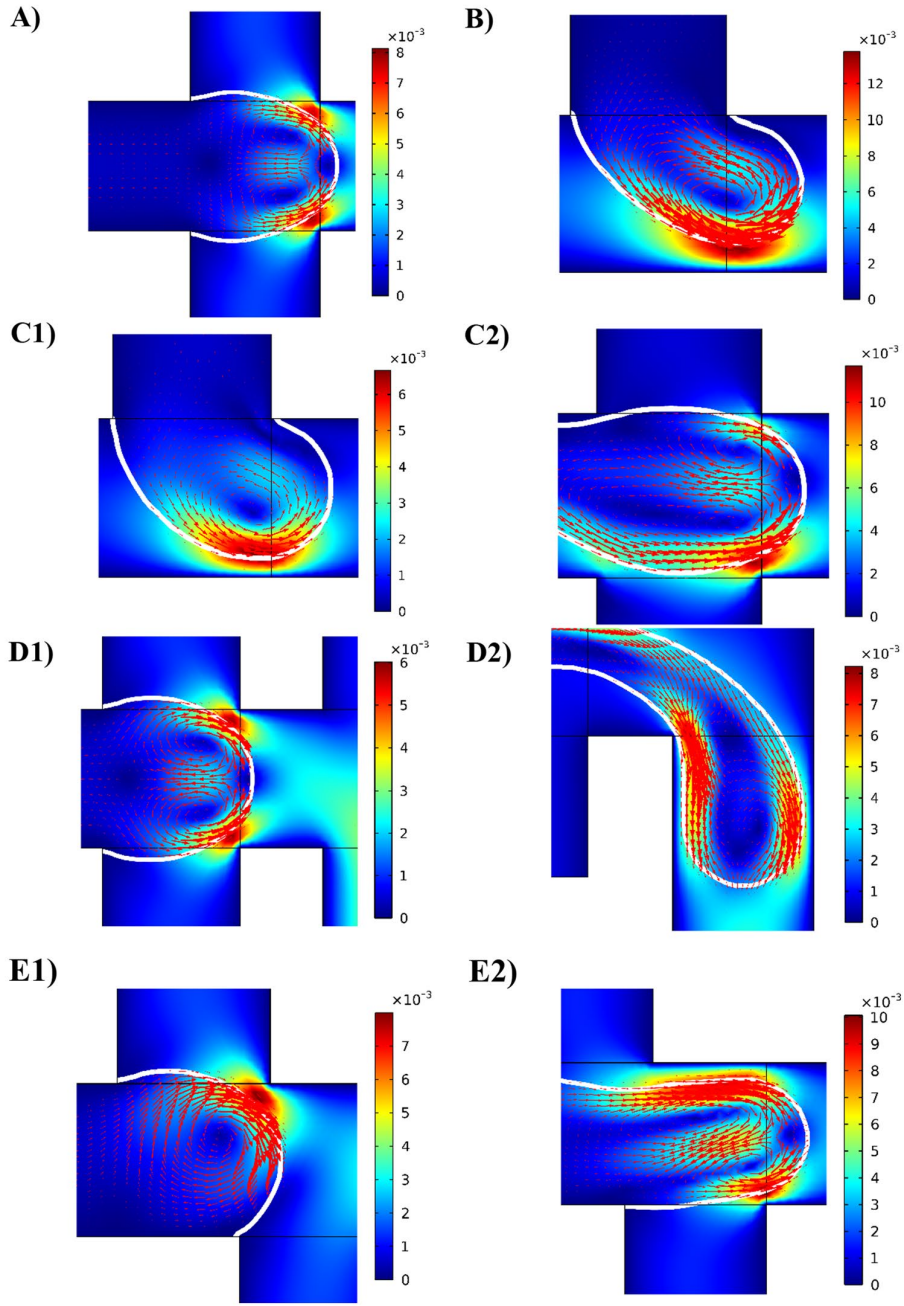
2D simulation of the droplet formation. Velocity distribution during the filling stage in (**A**) cross junction, (**B**) T junction, (**C1**) T junction of the T-cross, (**C2**) cross junction of the T-cross, (**D1**) cross junction of the cross-T, (**D2**) T junction of the cross-T, (**E1**) first junction of the asymmetric cross junction, and (**E2**) second junction of the asymmetric cross junction. The velocity ratio is 0.5, and color bars show the velocity magnitude in m/s.

Figure 2 demonstrates the geometries of droplet generators investigated in this research. In addition to the two well-known geometries, T junction and cross junction, three novel geometries are introduced in this study. The main idea behind these novel designs is combining the mixing mechanism and the recirculation vortex types of the cross junction and the T junction. Figure 4 illustrates that during the filling stage when the two immiscible fluids enter the main channel, the velocity of the interface is much lower than that of the continuous phase, resulting in the creation of one or two fluid recirculation vortices in the dispersed phase. These vortices are induced by the flow of the continuous phase due to the boundary conditions at the liquid–liquid interface. In the T junction, there is one large recirculation vortex during the filling stage, which mixes the species throughout the droplet, resulting in higher mixing efficiency compared to the cross junction. Conversely, in the cross junction, two symmetrical vortices exist, but they do not significantly contribute to the mixing process as they only mix the species in the two upper and lower halves of the droplet separately.

The T-cross (Fig. 4C) and cross-T (Fig. 4D) geometries take advantage of the mixing mechanisms of both the T junction and the cross junction. It is important to note that in the T junction, the vortex effectively mixes all the components inside the droplet (Fig. 4B). However, the velocity near the center of the vortex approaches zero, resulting in poor mixing efficiency in this area. On the other hand, in the cross junction, the two vortices in the droplet can mix the reagents in the center of the droplet and its sides by circulating the species from center to sides and vice versa (Fig. 4A). As a result, the two types of vortices in the T junction and the cross junction can complement each other's mixing performance, leading to higher mixing efficiency in the T-cross and cross-T geometries compared to the typical T and cross junctions.

In the asymmetric cross junction geometry (Fig. 4E), the offset between the two inlets of the continuous phase causes a delay in the formation of the two counter-circulating vortices in the cross junction. As the dispersed phase approaches the first inlet of the continuous phase, a single vortex forms inside the dispersed phase, similar to the T junction geometry. However, upon reaching the second continuous phase inlet, another vortex emerges within the dispersed phase with an opposite direction. At this point, the mixing mechanism becomes similar to that of the cross junction, as there are now two recirculation vortices. Therefore, it can be said that the asymmetric cross junction behaves similarly to the T-cross geometry.

### Droplet diameter and eccentricity

In many applications of droplet-based microfluidics, it is crucial to keep the created droplets away from the walls of the channels to avoid contamination of the droplets and their contents. Therefore, there is a high demand for producing small droplets. In addition to efficient mixing inside the droplets, the size and shape of the droplets are important factors to consider when comparing different droplet-generator designs.

**Figure 5:**
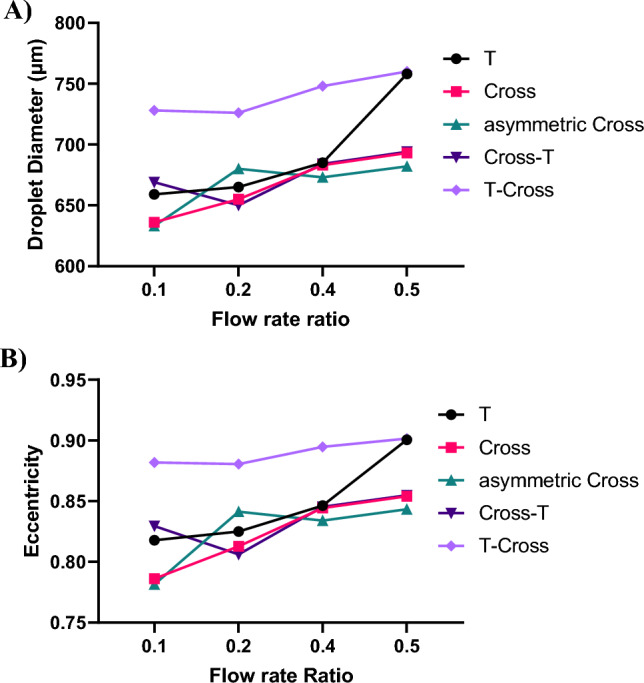
Diameter and eccentricity variations of droplets with flow rate ratio. Comparative analysis of (**A**) droplet diameter and (**B**) eccentricity in various geometries. The total flow rate of the continuous phase is $10 \mu l/min$.

Figure 5 illustrates the variations of droplet diameter and eccentricity as a function of the flow rate ratio of the dispersed phase to the continuous phase in different geometries. In this study, by considering droplets as ellipses, eccentricity was defined as $e = \sqrt {1 - b^{2} /a^{2} }$, where a and b represent the longest and the shortest diameters of the ellipse, respectively. The eccentricity value is used to gauge how much the droplet's shape differs from a perfect circle. Almost in all geometries, there is a consistent rise in the droplets' eccentricity as the flow rate of the dispersed phase increases. This happens because the larger droplet size exceeds the channel's width capacity, causing it to deform from its natural spherical shape. To be noted that the droplet diameter was calculated by considering the droplet as a circle.

When comparing these geometries, it becomes evident that the T-cross junction geometry exhibits the highest values for both droplet size and eccentricity, while the smallest values are observed in the cross junction and cross-T junction geometries. The novel geometries, asymmetric cross junction and cross-T junction, offer relatively small diameters in comparison to the T junction and comparable to the cross junction, which can be beneficial in many of applications. The T-cross junction produce the largest droplets. This is because the first junction in this geometry, T junction, is mainly responsible for the size of the droplet and the main shear force from the continuous phase is exerted on the dispersed phase in this junction. The second junction in this geometry, cross junction, has a more influential effect on the mixing efficiency since it determines the last recirculation vortex type inside the droplet.

In the contrary, in the cross-T junction, the first junction is the cross junction. As we expected, the produced droplets in this geometry are smaller than the T-cross junction. The second junction in this geometry, the T junction, makes a recirculation vortex as in the conventional T junction and increases the mixing efficiency.

In the asymmetric cross geometry, the pinch-off process and the droplet formation is similar to that in the conventional cross junction. Therefore, we expect the droplet size in this geometry to be around that in the cross junction. However, the offset between the two inlets of the continuous phase, increases the mixing efficiency inside the droplet due to the delay in the formation of the vortices inside droplets. Considering the droplet size and eccentricity in all geometries, the two novel geometries, cross-T junction and asymmetric cross junction, produce droplets with small sizes comparable to the conventional cross junction.

### Mixing efficiency

The mixing efficiency is important in many applications, and micromixers are commonly used in microfluidic systems to ensure proper mixing of substances within droplets. However, incorporating micromixers may require extra space. Additionally, instant mixing of substances upon contact can greatly enhance the consistency of chemical reactions and their resulting products. Given these factors, using a droplet generator that can mix substances within the droplet during the filling stage seems like a promising alternative to using micromixers.

**Figure 6:**
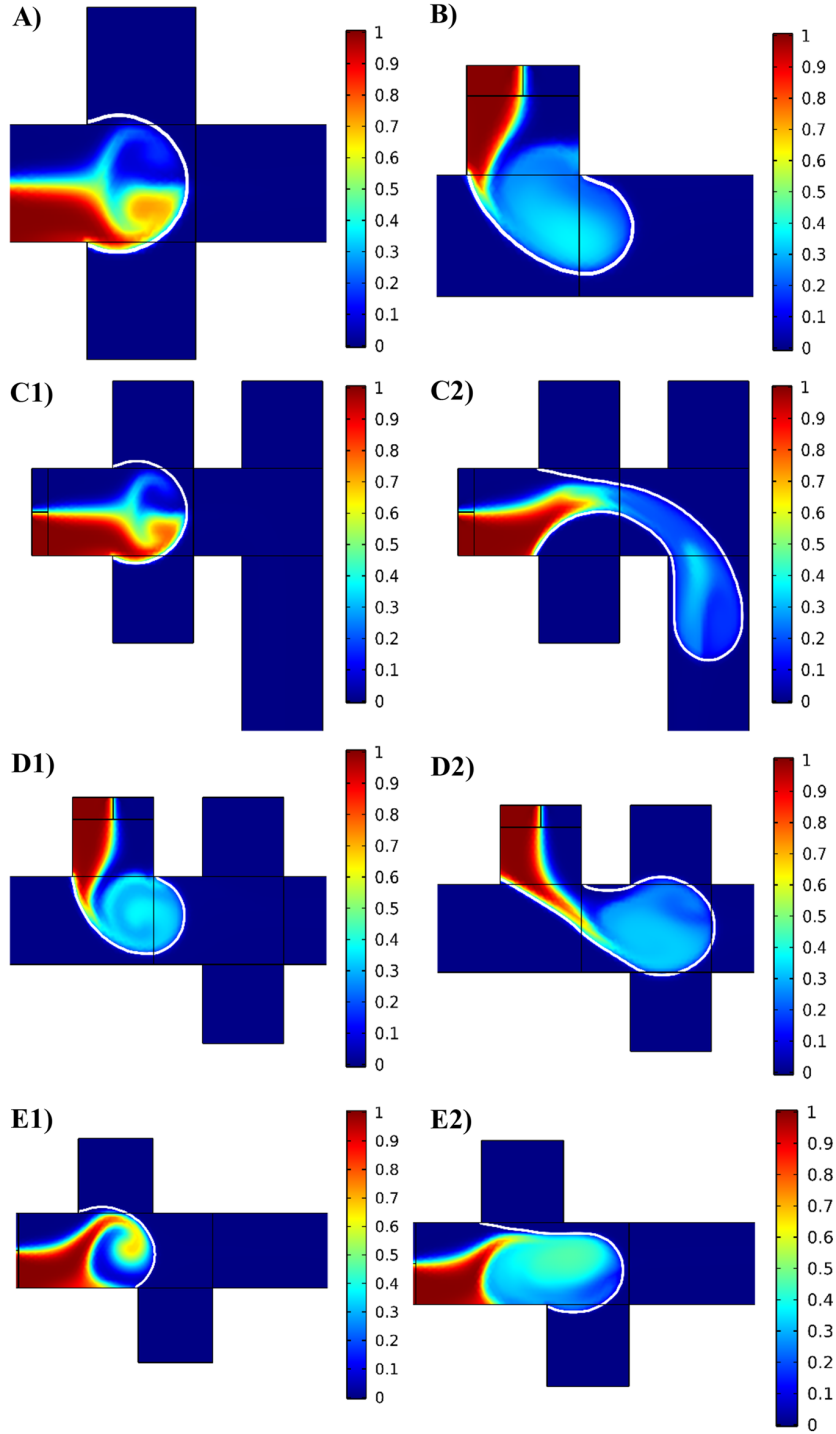
Mixing efficiency within droplets. Mixing efficiency in the filling stage in different junctions: (**A**) cross junction, (**B**) T junction, (**C1**) cross junction of the cross-T, (**C2**) T junction of the cross-T, (**D1**) T junction of the T-cross, (**D2**) cross junction of the T-cross, (**E1**) first junction of the asymmetric cross junction, and (**E2**) second junction of the asymmetric cross junction. Continuous phase flow rate is $10 {\mu l}/{\text{min}}$ and the flow rate ratio is 0.2 for all cases. The color bars demonstrate the relative dye concentration.

Figure 6 presents the visualization of the efficiency of mixing within the dispersed phase in the filling stage in various junctions. It is important to note that the process of droplet formation and the characteristics of recirculation vortices inside the droplets significantly influence the mixing efficiency. Apparently, novel geometries demonstrate considerable mixing in comparison to the conventional T and cross junctions.

**Figure 7:**
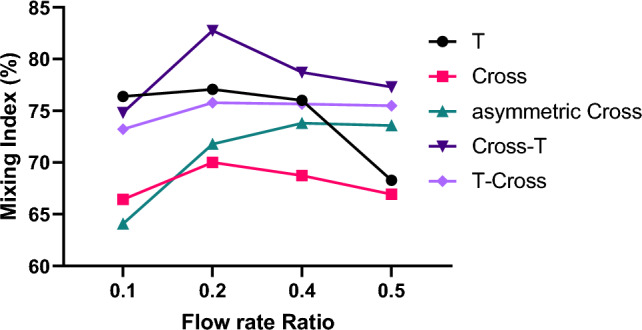
Mixing efficiency variations within droplets with flow rate ratio in different geometries. Comparative analysis of mixing in various Geometries. The overall flow rate of the continuous phase is $10 \mu l/min$.

Figure 7 illustrates the quantified analysis of the mixing index in the droplets right after the droplet formation in various geometries. Almost in all geometries, a consistent trend is observed: as the flow rate of the dispersed phase increases, the mixing efficiency decreases. This phenomenon can be explained by the reduction in filling stage duration that occurs when the dispersed phase flow rate is increased. This implies that the substances inside the droplets do not have enough time for thorough mixing due to the presence of formed vortices. However, in asymmetric cross geometry, the trend is reverse.

The cross-T junction geometry exhibits the highest mixing efficiency due to the existence of both types of vortices: two counter-circulating in the cross junction and one vortex in the T junction of this geometry. These two types of vortices complement each other, leading to a high mixing efficiency. This innovative geometry increases the mixing efficiency by approximately 10% compared to the conventional T junction, which offers the best mixing efficiency among conventional droplet generators. It is worth noting that there is an increase in the mixing efficiency while the size of droplet in the cross-T is smaller than in the T junction.

The mixing index is lower in the T-cross geometry compared to the cross-T. This discrepancy arises because the first junction in these geometries primarily influences droplet size, and the primary shear force from the continuous phase acts on the dispersed phase in this junction. In these geometries, the second junction plays a more crucial role in mixing efficiency as it dictates the final recirculation vortex type within the droplet, and this vortex also persists for a longer time. As a result, in the cross-T geometry, the size of the droplet is determined by the cross junction, and the last mixing vortex, responsible for mixing all analytes throughout the entire droplet, is determined by the T junction. Consequently, the cross-T geometry yields smaller-diameter droplets with higher mixing indices compared to the T-cross geometry.

In the asymmetric cross geometry, the offset between the two continuous phase inlets results in the geometry behaving like two distinct T junctions, generating two counter-rotating vortices in the dispersed phase. These consecutive vortices enhance the mixing index compared to the cross junction. However, compared to the cross-T and T-cross geometries, the asymmetric cross geometry lacks two distinct mixing mechanisms (cross and T junction mixing vortices), preventing it from achieving an increased mixing index like the cross-T and T-cross geometries.

However, all innovative geometries demonstrate comparable mixing efficiency compared to the T junction. The cross junction exhibits the lowest mixing efficiency due to the symmetry in this geometry, where the two flows of the continuous phase are identical. As a result, two identical vortices but in opposite directions are formed inside the droplet, which only mix the substances in the upper and lower halves of the droplet separately. Because there is no single vortex enveloping the entire droplet, this unique configuration leads to the lowest mixing efficiency values among all the geometries.

## Conclusions

In this research, three novel droplet generator designs that offer high mixing indices and small droplet sizes are introduced: T-cross, cross-T, and asymmetric cross geometries. The T-cross and cross-T geometries combine cross and T junction mixing mechanisms, resulting in increased mixing efficiency. Numerical simulations were conducted and these novel geometries were compared with traditional T and cross junctions in terms of mixing index, droplet diameter, and eccentricity. In all cases, the overall flow rate of the continuous phase was considered the same ($10 \mu l/min$) to have identical conditions for all geometries. The cross-T geometry had the highest mixing index and produced the smallest droplets. At a flow rate ratio of 0.5, this design provided a 10% enhancement in the mixing index and a 10% reduction in droplet diameter compared to the T junction. While the T junction has the best mixing efficiency among traditional droplet generators, it produces larger droplets, which can increase the risk of contamination due to the contact of the droplet with walls of the microchannel. The cross-T geometry is therefore highly desirable in most applications since produces droplets with considerably small droplets. In a flow rate ratio of 0.5, the asymmetric cross junction demonstrated an 8% improvement in the mixing index and approximately a 2% reduction in droplet diameter compared to the standard cross junction. All novel geometries showed comparable mixing efficiency to the T junction, with droplet sizes between those of the cross and T junctions. The cross junction had the lowest mixing efficiency and droplet diameter larger than the cross-T geometry. Therefore, the novel geometries, particularly the cross-T geometry, are a favorable choice for applications where both high mixing efficiency and smaller droplet sizes are important.

In the future, I plan to investigate the impact of offset size on the mixing index in the asymmetric cross junction. Additionally, studying the distance between the T and cross junctions in the T-cross and cross-T geometries would be beneficial.

## Data Availability

The datasets generated and/or analyzed during the current study are not publicly available due to requirement of confidentiality, but are available from the corresponding author on reasonable request.
